# Cashew Nut Shell Waste Derived Graphene Oxide

**DOI:** 10.3390/molecules29174168

**Published:** 2024-09-03

**Authors:** Alvaro Arrieta, Yamid E. Nuñez de la Rosa, Samuel Pestana

**Affiliations:** 1 Department of Biology and Chemistry, Universidad de Sucre, Sincelejo 700001, Colombia; samuel.pestana@udea.edu.co; 2 Faculty of Engineering and Basic Sciences, Fundación Universitaria Los Libertadores, Bogotá 111221, Colombia; yenunezd@libertadores.edu.co

**Keywords:** graphene oxide, cashew nut shell, modified Hummers method, sustainability

## Abstract

The particular properties of graphene oxide (GO) make it a material with great technological potential, so it is of great interest to find renewable and eco-friendly sources to satisfy its future demand sustainably. Recently, agricultural waste has been identified as a potential raw material source for producing carbonaceous materials. This study explores the potential of cashew nut shell (CNS), a typically discarded by-product, as a renewable source for graphene oxide synthesis. Initially, deoiled cashew nut shells (DCNS) were submitted to pyrolysis to produce a carbonaceous material (Py-DCNS), with process optimization conducted through response surface methodology. Optimal conditions were identified as a pyrolysis temperature of 950 °C and a time of 1.8 h, yielding 29.09% Py-DCNS with an estimated purity of 82.55%, which increased to 91.9% post-washing. Using a modified Hummers method, the Py-DCNS was subsequently transformed into graphene oxide (GO-DCNS). Structural and functional analyses were carried out using FTIR spectroscopy, revealing the successful generation of GO-DCNS with characteristic oxygen-containing functional groups. Raman spectroscopy confirmed the formation of defects and layer separations in GO-DCNS compared to Py-DCNS, indicative of effective oxidation. The thermogravimetric analysis demonstrated distinct thermal decomposition stages for GO-DCNS, aligning with the expected behavior for graphene oxide. Scanning electron microscopy (SEM) and energy dispersive X-ray spectroscopy (EDX) further corroborated the morphological and compositional transformation from DCNS to GO-DCNS, showcasing reduced particle size, increased porosity, and significant oxygen functional groups. The results underscore the viability of cashew nut shells as a sustainable precursor for graphene oxide production, offering an environmentally friendly alternative to conventional methods. This innovative approach addresses the waste management issue associated with cashew nut shells and contributes to developing high-value carbon materials with broad technological applications.

## 1. Introduction

Graphene oxide (GO) has attracted the scientific community’s attention due to its exceptional properties and broad application potential in various technological fields. This substance, composed of a sheet of functionalized graphene with oxygenated groups on its surface, exhibits a two-dimensional structure of carbon atoms and oxygen-containing functional groups on both sides and edges of the carbon plane [1]. The first works on the synthesis of graphene oxide from exfoliated graphite oxide were reported in 1859 by Benjamin Brodie [1,2]. However, Novoselov and Geim reported the discovery of graphene and its properties in 2004, for which they were awarded the Nobel Prize in Physics in 2010 and popularized graphene and its derivatives [3,4]. Although graphene oxide shares the characteristic of being a two-dimensional carbon material, its properties are remarkably different from those of graphene. Graphene oxide exhibits more pronounced chemical activity compared to graphene. It has thus attracted attention due to its versatility and ability to be functionalized and modified with a variety of chemical groups, which broadens its applicability in diverse fields.

Graphene oxide has emerged as a cutting-edge material with revolutionary potential in a wide range of technological applications. For example, in the field of electronics, the use of graphene oxide deposited on graphite sheets as an intermediate layer to make polymer light-emitting diodes (PLED) has been reported [5,6]. In addition, graphene oxide synthesized via chemical method from commercial graphite has been used in the development of photonic and electronic integrated circuits [7,8] and in information storage [9,10]. It has also been reported that an absorber with a tunable bandwidth and polarization independence, utilizing monolayer graphene, can achieve an absorption rate exceeding 0.9 in the 3–6 THz range [11]. A photoelectric in-fiber device has also been proposed using few-layer graphene wrapped around a tilted fiber Bragg grating (TFBG) with bonded electrodes for photoelectric and thermo-optic conversions [12]. Furthermore, a review was conducted on the advancements in graphene-based dynamic metasurfaces and metadevices, emphasizing the electrically controlled manipulation of EM waves across the mid-infrared, terahertz, and microwave ranges [13].

Its electrical conductivity and its large specific surface offer significant advantages over other materials used in these devices. Additionally, graphene oxide has shown great potential in the development of devices related to energy storage and generation, such as solar cells, batteries and supercapacitors, due to its ability to facilitate the rapid transport of electrons and ions, so several reviews have been published on these applications [14,15,16,17]. Additionally, the technological use of graphene oxide has been reported in its application in regenerative medicine due to the compatibility and ability to form hydrazine-rGO, ginseng-rGO complexes, which allowed the accelerated differentiation of neural stem cells [18]. Promising applications have also been explored in cancer therapies, diagnostic imaging and tissue regeneration, among others [19,20,21,22,23].

Due to its technological potential, it is estimated that the demand for graphene oxide will increase dramatically in the coming years, which has facilitated the intensification of work aimed at developing synthesis methods that allow obtaining this material in large quantities and by economically viable methods [24,25,26]. In their review, Edward et al. (2023) [27] present a detailed table describing various graphene synthesis methods. Each process is outlined in terms of the techniques employed, the characteristics of the final product, and its potential applications. Additionally, the table offers a comparative analysis of the advantages and disadvantages of each method, providing a clear and comprehensive view of the options available for graphene production, tailored to the specific needs of each industrial or research application. In parallel to the search for new synthesis methods for graphene oxide, the exploration of alternative and sustainable raw materials has gained relevance in recent years. In this context, agricultural waste has been identified as a potentially rich and underutilized source of raw materials for the production of carbonaceous materials. The use of rice husk to obtain graphene and reduced graphene oxide by applying the Hammers method has been reported [28,29]. Coconut shell is another bio-waste used to obtain graphene and graphene oxide from its carbonization and transformation by oxidation with sodium nitrate, sulfuric acid and potassium permanganate [30,31]. Corn cob has also been used to obtain graphene oxide by applying the modified Hummers method [32,33].

On the other hand, cashew nut shells, a commonly discarded by-product in the food industry, have emerged as an attractive option due to their abundance, low cost and favorable chemical composition. Between 2015 and 2021, global raw cashew production experienced a remarkable increase, reaching a total of 24.37 million tons. Therefore, the annual cashew production is estimated to be approximately 4 million tons, considering that the weight ratio of the nut to the shell is approximately 1:2.5, the cashew shell production is estimated at 10 million tons [34]. Shells are generally discarded as waste, since they currently have no commercial value, so they are discarded in production environments or burned in the open air. This creates an environmental problem mainly due to the oil content of the shells or cashew nut shell liquid (CNSL), which can harm human health and soils when not managed correctly. CNSL is composed of anacardic acid, cardanol and cardol and is the subject of numerous investigations due to its chemical properties and because it has shown to be a promising source for the generation of new materials, mainly polymeric [35,36,37,38]. However, after the CNSL extraction process, the squeezed shell remains, which can be used as a source of carbon due to its chemical composition, which includes lignin, cellulose, hemicellulose and other polysaccharides, which provides a suitable starting point for obtaining carbonaceous materials such as graphene oxide.

Cashew nut shell has been reported to be used to obtain activated carbon by chemical activation with KOH and to obtain carbon with high surface area (2742 m^−2^ g^−1^) and porosity (1.528 cm^3^ g^−1^) to make a supercapacitor with an energy density of 11.2 to 400 Wh kg^−1^ and a capacitive efficiency of 305.2 F g^−1^ [39]. In addition, cashew nut shell activated carbon has been obtained by KOH activation to adsorb bright green dye [40] and phosphoric acid activated carbon has also been prepared from cashew nut shell modified by grafting polyethylenimine to remove Cr (VI) [41]. However, no reports were found on obtaining graphene oxide or graphene materials from cashew nut shells. As mentioned above, most research has explored different approaches for producing graphene oxide from renewable resources such as rice husk, corn cob, and coconut shells. This work evaluates the potential of deoiled cashew nut shell biowaste as a renewable source for graphene oxide synthesis and its structural and functional properties.

## 2. Results and Discussion

**Figure 1:**
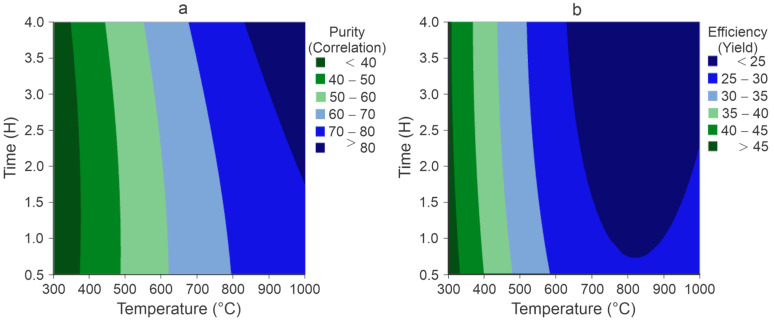
Contour plot of (**a**) Py-DCNS purity vs. pyrolysis time and temperature and (**b**) Py-DCNS efficiency vs. pyrolysis time and temperature.

Cashew nut shells (CNS) were squeezed to obtain deoiled shells (DCNS). These DCNS were used to generate carbonaceous material by pyrolysis (Py-DCNS). The pyrolysis process was optimized to achieve higher purity and yield. For this purpose, a response surface experiment design was applied where the input variables were the pyrolysis time and temperature and the output variables were the yield (measured in percentage from the g of Py-DCNS obtained and the g of DCNS used; (*g Py*-*DCNS/g DCNS*) × 100) and purity (estimated by FTIR correlation of Py-DCNS with commercial graphite of 99.99% purity). Figure 1 shows the contour graphs of purity (Figure 1a) and yield (Figure 1b). It can be seen in Figure 1a that temperature is a factor that directly affects purity, presenting a lower degree of purity (correlations less than 0.7) at temperatures below 800 °C and correlations above 0.7 and 0.8 around 900 °C and more significant than 0.8 at approximately 1000 °C. It is also observed that the pyrolysis time does not show a marked effect on purity at pyrolysis temperatures lower than or close to 700 °C and shows a greater influence between 700 and 1000 °C, presenting a greater correlation in pyrolysis times between 2 and 4 h.

Additionally, it can be seen in Figure 1b that temperature significantly affects the production yield of Py-DCNS, showing the highest values (between 45% and 35%) at temperatures between 300 and 600 °C and yields less than 35% at temperatures higher than 600 °C, with 25% as the lowest value obtained between 900 and 1000 °C. It is also evident that time is not a relevant factor on the yield of the pyrolysis process at temperatures below 700 °C, while at temperatures between 700 and 1000 °C it shows an antagonistic effect; at approximately 800 °C the yield decreases to less than 0.25 when pyrolysis times are greater than 0.7 h, while at 700 °C this time is 1.5 h and at 1000 °C it is 2 h.

**Figure 2:**
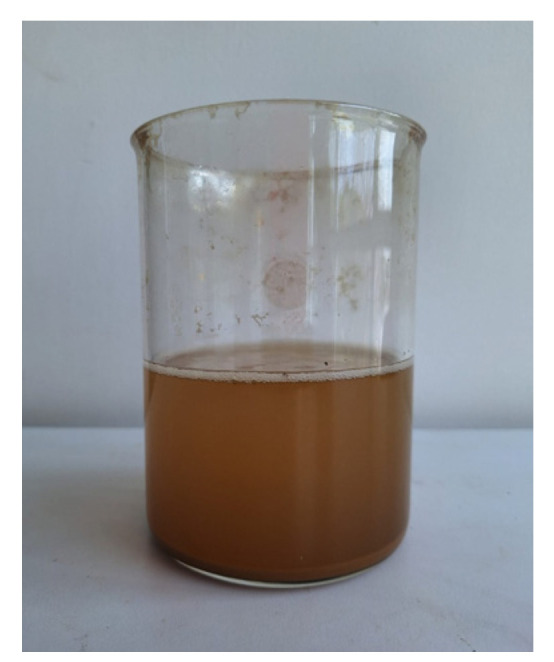
GO-DCNS dispersed in water by ultrasonication.

The observed behavior in yield and purity in terms of pyrolysis time and temperature may be because at low temperatures, complete carbonization does not occur, so the amount of burned material is lower and therefore, a higher yield is obtained because there may be uncarbonized remaining material. Thus, the purity of the carbonaceous material tends to be lower at low pyrolysis temperatures. At higher temperatures (between 700 and 1000 °C), all the material and the substances that compose it are carbonized, resulting in greater purity, but lower yield. On the other hand, time does not show an appreciable effect on yield or purity at low temperatures. At low temperatures, incomplete pyrolysis occurs and is reached quickly, so it is not significantly affected by extending the time. At temperatures above 700 °C, longer pyrolysis times are necessary because a more complete carbonization and thermal degradation develop, thus being affected by the time used in the process. The optimization model aimed at maximizing the yield and purity of Py-DCNS yielded an optimal temperature of 950 °C and an optimal pyrolysis time of 1.8 h with an R-sq of 94.2%. Under these conditions, a yield of 29.09% and an estimated purity by correlation of 82.55% were obtained. The Py-DCNS was washed to remove impurities from the pyrolysis process and purity values of approximately 91.9% were achieved. Using the modified Hummers method, the carbonaceous material (Py-DCNS) was generated from this optimization process at 950 °C and 1.8 h synthesize graphene oxide (GO-DCNS). Figure 2 shows an image of the GO-DCNS obtained through the synthesis process and dispersed in water by ultrasonication. As mentioned above, the yield of the carbonization process was 29.09%; 0.2909 g of carbonaceous material (Py-DCNS) were produced from one gram of deoiled shell (DCNS). On the other hand, 0.9091 g of GO-DSCN were produced from one gram of carbonaceous material, which reflects a yield of the synthesis process of 90.91%. Thus, the yield of the process for obtaining GO-DCNS from the initial DCNS material was 26.29%, with an energy consumption of approximately 0.48 kWh per gram of GO-DCNS.

**Figure 3:**
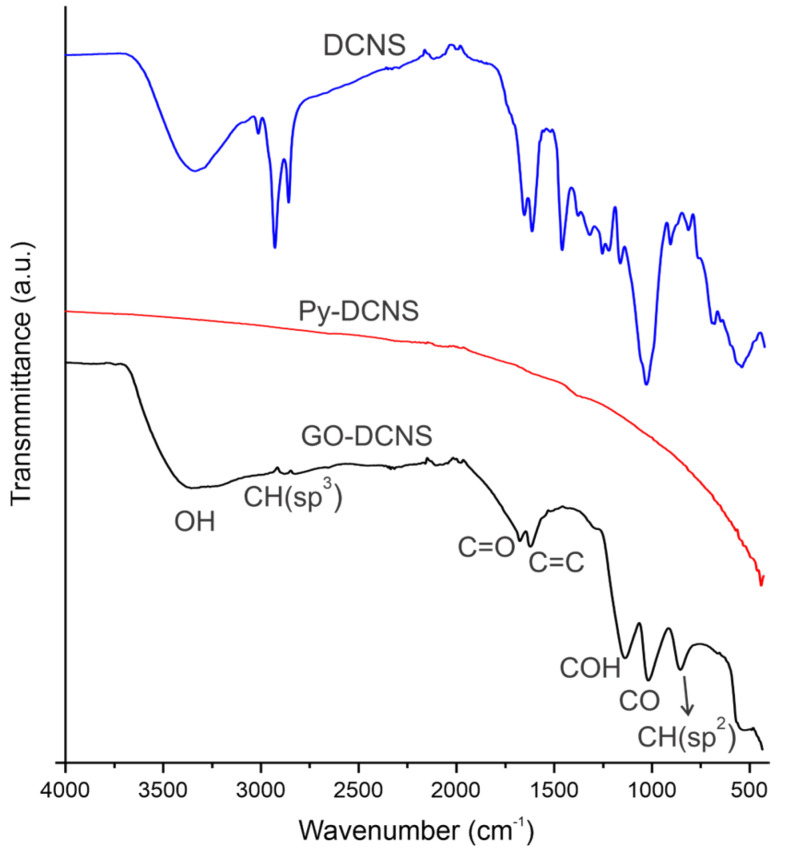
FTIR spectra of DCNS, Py-DCNS and GO-DCNS.

The structural changes that occurred during the transformation of DCNS to Py-DCNS and during the conversion of Py-DCNS to graphene oxide (GO-DCNS) were analyzed by FTIR spectroscopy. In Figure 3, the FTIR spectra of DCNS, Py-DCNS and GO-DCNS are presented and the bands observed in the spectra corresponding to the characteristic vibrations of each one is summarized in Table 1.

The FTIR spectrum of DCNS shows several functional groups characteristic of CNSL; a broad band at 3334 ${cm}^{-1}$ and another at 1244 ${cm}^{-1}$ due to the vibration of the OH groups, the vibration of the functional group =CH (${sp}^{2}$) at 3009 ${cm}^{-1}$, while at 2924 and 2854 ${cm}^{-1}$ the band assigned to the vibration −CH is observed. The bands at 1646 ${cm}^{-1}$ are assigned to the carboxylic acid’s C=O vibrations, and 1604 and 1450 ${cm}^{-1}$ correspond to O-H and C-H vibrations, respectively. The CO bond vibrations are seen at 1027 ${cm}^{-1}$. The presence of the characteristic functional groups of CNSL in the DCNS spectrum is because, although the shells were subjected to an oil extraction process, some remains of the CNSL components, such as anacardic acid, cardanol and cardol remain trapped in the deoiled shells.

On the other hand, the FTIR spectrum of Py-DCNS does not show any defined band due to the chemical inertia of this sample. On the other hand, the graphene oxide (GO-DCNS) spectrum obtained from Py-DCNS by the modified Hummers method, presents vibrations of the functional groups produced by oxidation, with bands at 3356, 1719, 1149 and 1030 ${cm}^{-1}$ generated by the vibration of the OH groups, the C=O carboxylic bonds, the vibrations of the C-OH bonds and those corresponding to the C-O vibrations, respectively. Additionally, weak bands are observed at 2951 and 2906 ${cm}^{-1}$ due to the CH (${sp}^{2}$) and CH (${sp}^{3}$) vibrations and the CH (${sp}^{2}$) group vibration at 867 ${cm}^{-1}$.

The chemical inertness observed in the spectrum of Py-DCNS confirms the effectiveness of the pyrolysis process in the carbonization of DCNS and the presence of functional groups containing oxygen and the vibrations associated with the aromatic rings are evidence of the generation of graphene oxide (GO-DCNS). These results agree with those previously reported by other authors concerning the production of graphene oxide [42,43].

**Figure 4:**
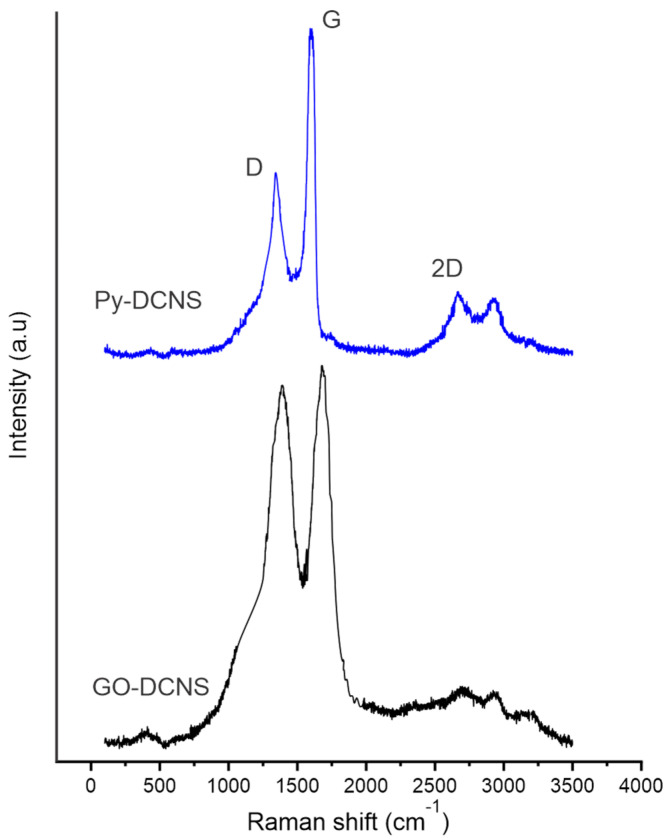
Raman spectrum of pyrolyzed deoiled cashew nut shell (Py-DCNS) and graphene oxide synthesized from pyrolyzed deoiled cashew nut shell (GO-DCNS).

The production of graphene oxide from Py-DCNS was studied by Raman spectroscopy. The Raman spectra of Py-DCNS and GO-DCNS are shown in Figure 4. The characteristic D, G and 2D bands are observed in the spectra of both compounds. The Raman spectrum of Py-DCNS shows an intense G band located at 1584 ${cm}^{-1}$ associated with the laminar structure of carbonaceous material, composed of multiple stacked layers of graphene with ${sp}^{2}$ hybridization, a D band located at 1321 ${cm}^{-1}$ of lower intensity due to the low number of defects and structural vacancies. Additionally, the 2D band with moderate intensity originated by a high number of graphene layers is observed at 2684 ${cm}^{-1}$.

The spectrum of graphene oxide (GO-DCNS) presents a more intense D band compared to the spectrum of the pyrolyzed shell (Py-DCNS), due to the formation of functional groups produced by oxidation and chemical transformation that generates defects, distortions and vacancies in the structure. The generation of defects due to the formation of oxygen functional groups occurs in the π orbitals (C=C), promoting the conversion of ${sp}^{2}$ hybridized orbitals into ${sp}^{3}$, which is reflected in the increase in intensity in the D band in the GO-DCNS spectrum. In addition, this phenomenon is also related to the broadening and shift of the D band to a slightly higher wavelength value (1335 ${cm}^{-1}$) about that observed in the Py-DCNS spectrum. The G band appears at 1599 ${cm}^{-1}$, showing a slight shift, lower intensity and greater width than in Py-DCNS. These changes in the G band are due to a higher resonance frequency of the isolated double bonds in the structure of the individual layers of graphene oxide. On the other hand, the 2D band observed at 2691 ${cm}^{-1}$ is not very intense in the spectrum of GO-DCNS, due to the low number of layers of the graphene oxide obtained, while the pyrolyzed material shows a more intense 2D band due to the greater number of layers in its structure.

The relationship between D and G bands has been widely recognized as a method to elucidate the structural organization of carbonaceous materials [44]. Thus, the observed differences are accompanied by the increase in the ratio of intensities in the D and G bands (i.e., ID/IG), which for Py-DCNS is 0.34 and 0.98 for GO-DCNS, indicating a reduction in the size and crystallinity of the carbonaceous material due to oxidative chemical transformation and layer separation. This is consistent with previously reported findings [45].

**Figure 5:**
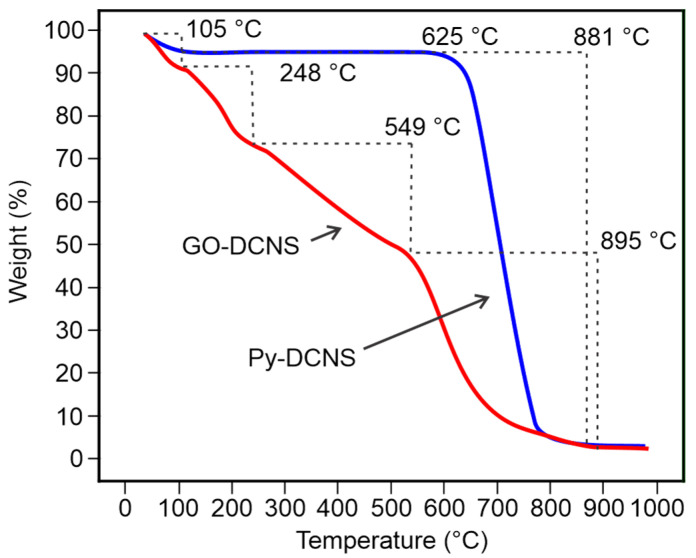
Thermogravimetric analysis (TGA) of Py-DCNS (blue) and GO-DCNS (red).

The thermal decomposition analysis performed by TGA is presented in Figure 5. It can be observed that the GO-DCNS shows four well-defined thermal decomposition processes. An initial process due to dehydration by loss of water molecules represents the 7% observed in the temperature range from room temperature to 105 °C; the humidity of the material can be due to water adsorption during the synthesis. The second thermal decomposition process takes place from 105 °C to 238 °C, with a weight loss of 18% due to the decomposition and elimination of the labile functional groups produced during the oxidation process in the form of CO_2_ and CO. 

The third process is shown between 238 and 549 °C, with an approximate weight loss of 26% due to the degradation of the most stable oxygenated functional groups. Finally, a fourth stage of more marked decomposition due to gasification and sublimation of the carbon structure of GO-DCNS with an approximate weight loss of 48% occurs between 549 and 895 °C. The thermogravimetric behavior of Py-DCNS is similar to that reported for graphite [46]. A small weight loss of 3% is observed between the start of heating (30 °C) and 105 °C due to dehydration and a massive thermal decomposition process between 625 and 881 °C corresponding to a weight loss of approximately 94%, attributed to the degradation of the graphemic carbon skeleton [46].

**Figure 6:**
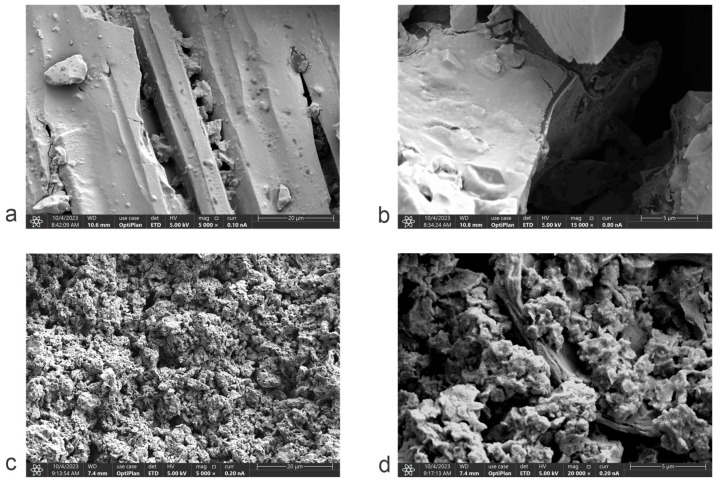
Scanning electron microscopy of Py-DCNS samples with (**a**) 5 k scale and (**b**) 20 k scale and GO-DCNS samples with (**c**) 5 k scale and (**d**) 20 k scale.

The microstructure of the carbonaceous material produced by the pyrolysis of deoiled cashew nut shells (Py-DCNS) and the graphene oxide synthesized from it (GO-DCNS) was analyzed by scanning electron microscopy (SEM) at two magnifications of 5 k and 20 k. The micrographs of Py-DCNS are presented in Figure 6a (5 k) and Figure 6b (20 k) and those of GO-DCNS in Figure 6c (5 k) and Figure 6d (20 k). The micrographs show a morphological difference between Py-DCNS and GO-DCNS. The carbonaceous material obtained from the pyrolysis of the deoiled shell shows at 5 k magnification, a characteristic shape of wide and coarse particles with a compact structure and sharp corners. While at 20 k magnification, a lamellar morphology indicating a layered structure in Py-DCNS is visible. On the other hand, the 5 k micrographs of graphene oxide show a change in morphology generated by the chemical transformation process of oxidation and generation of functional groups with oxygen and exfoliation promoted by the application of ultrasound, with a smaller particle size and aggregates. The magnification at 20 k allows us to appreciate a porous structure with sheets with roughness produced by possible distortions of the graphene sheets during oxidation; the stabilization of the atomic arrangement at the edges of the sheets forms the wrinkles.

**Figure 7:**
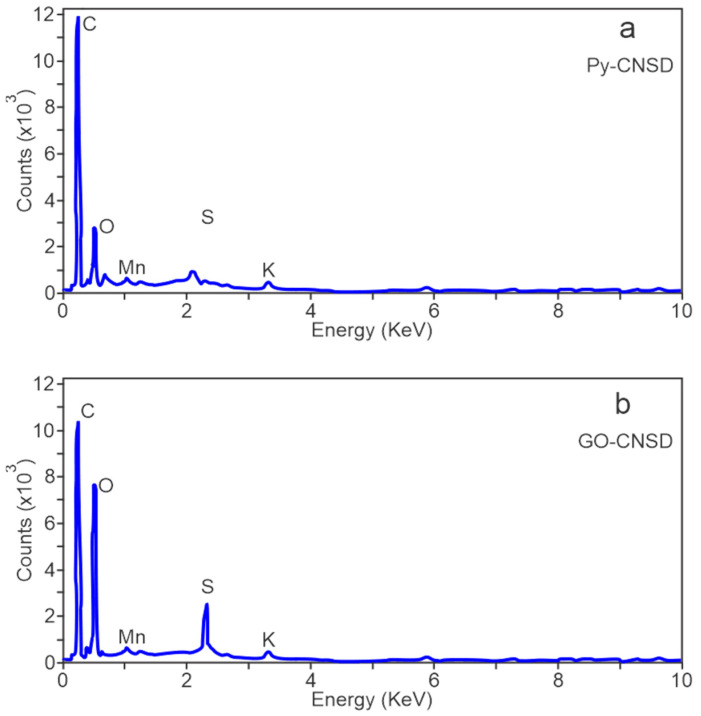
EDX spectra (**a**) Py-DCNS and (**b**) GO-DCNS.

The morphological differences between Py-DCNS and GO-DCNS show a transformation due to chemical oxidation and exfoliation during synthesis. Scanning electron microscopy is generally combined with energy dispersive X-ray spectroscopy (EDX), which allows for obtaining a map of the elemental composition of the samples in a specific area. Figure 7 presents the EDX spectra of the deoiled and pyrolyzed cashew nut shell (Py-DCNS) and the graphene oxide synthesized from carbonaceous material obtained from pyrolysis (GO-DCNS). It can be observed that the measurements carried out by EDX on both materials showed significant differences in their composition. The weight and atomic percentages of the elements detected by EDX in the Py-DCNS and GO-DCNS samples are presented in Table 2.

In the EDX spectrum of Py-DCNS (Figure 7a), the highest intensity of the carbon atom at 0.28 KeV is due to the structure of carbonaceous material with a percentage of 85.5% and oxygen in a lesser amount at 0.45 KeV with a percentage of 8.3%. Additionally, some elements are observed in smaller amounts, such as K, Co, S, and Na, with percentages of 1.6%, 1.4%, 2.1% and 1.1%, respectively. These elements present in Py-DCNS are possibly remnant elements from the pyrolysis process of the deoiled cashew nut shell. The EDX spectrum of GO-DCNS (Figure 7b) shows O (oxygen) and C (carbon) atoms at approximately 0.45 and 0.28 KeV, respectively, with an evident decrease in the intensity corresponding to carbon and an increase in the intensity assigned to oxygen relative to Py-DCNS, with a weight percentage of 53.7% for carbon and 40.8% for oxygen.

The intensity changes are mainly due to the functional groups generated in the graphene oxide structure (GO-DCNS) by the oxidation of Py-DCNS carbonaceous material by adding oxidizing agents used during its synthesis. Also, the addition of oxidants such as sulfuric acid and other oxidizing agents during the synthesis can eliminate Co and Na atoms, increase the presence of sulfur and generate the presence of the element Mn added in the synthesis process.

## 3. Materials and Methods

### 3.1. Reagents and Equipment

Reagents were purchased from Merck (Darmstadt, Germany) and Sigma-Aldrich (st. Louis, MO, USA). Ultrapure water was used to prepare the solutions and carry out the reactions. The reagents used were; sulfuric acid (98.08%, Merck), hydrochloric acid (37%, Aldrich), sodium hydroxide (99.99%, Merck), sodium nitrate (99.95, Merck), potassium permanganate (99.0%, Aldrich), hydrogen peroxide (30.0%, Merck) and graphite (99.99%, Aldrich). The cashew nut shell used was from Anacardium occidentale Yucao Ao3 variety provided by the company ASOPROMARSAB (Cordoba, Colombia).

The analysis by FTIR spectroscopy was carried out using a Perkin Elmer ATR (attenuated total reflectance) spectrometer, reference IR Spectrum two (Waltham, MA, USA). The thermogravimetric analysis was performed with a Perkin Elmer thermal analyzer, reference STA 6000 (Waltham, MA, USA). The Raman spectroscopy was performed with a LabRam spectrometer, reference HR800UV of Horiba Scientific (Irvine, CA, USA). Scanning electron microscopy with an energy dispersive detector (SEM-EDX) reference JSM 5610 from JEOL (Tokyo, Japan) was used to record the micrographic images and EDX spectra. Analytical balance (reference Practum) and micro balance (reference Secura) from Satorius were employed for weighing tasks (Goettingen, Germany). Eppendorf pipettes were used to handle liquids (Eppendorf SE, Hamburg, Germany). An MM5 Multipurpose Muffle (5 L) 1200 degrees from Terrigeno (Medellín, Colombia) was used for pyrolysis.

### 3.2. Obtaining Carbonaceous Material from Deoiled Cashew Nut Shells

Cashew nut shells (CNS) were collected from the association Asopromarsab (Chinú, Córdoba, Colombia). Once transported to the laboratory, they were washed and dried in an oven at 80 °C for 36 h before use. Once dried, the oil was extracted by mechanical pressing at room temperature using an electric press mill. The shell oil (CNSL) and the deoiled nut shell (DCNS) were obtained from the deoiling process. The CNSL was stored for another research, while the DCNS was crushed to be pyrolyzed in a muffle furnace. The pyrolysis process was optimized by developing a response surface experiment design using as input variables the temperature from 300 to 1000 °C and the time from 0.5 to 4 h; as output variables the yield (determined by gravimetry) and the purity (estimated by FTIR correlation with high purity commercial graphite). For this purpose, 50 g of DCNS were placed in a crucible, which was covered with ceramic fiber and sealed. Once the pyrolysis was finished, the material was allowed to cool to room temperature and was washed to remove traces of ash and contaminants. 

The washing was carried out with 10 mL of 37% HCl by sonication for 3 h at 45 °C, then it was washed with 20 mL of deionized water and NaOH was added drop by drop until reaching a pH of 6.5, then washed again with water and dried for 24 h at 100 °C and sieved.

### 3.3. Synthesis of Graphene Oxide from Deoiled and Pyrolyzed Cashew Nut Shell

**Figure 8:**
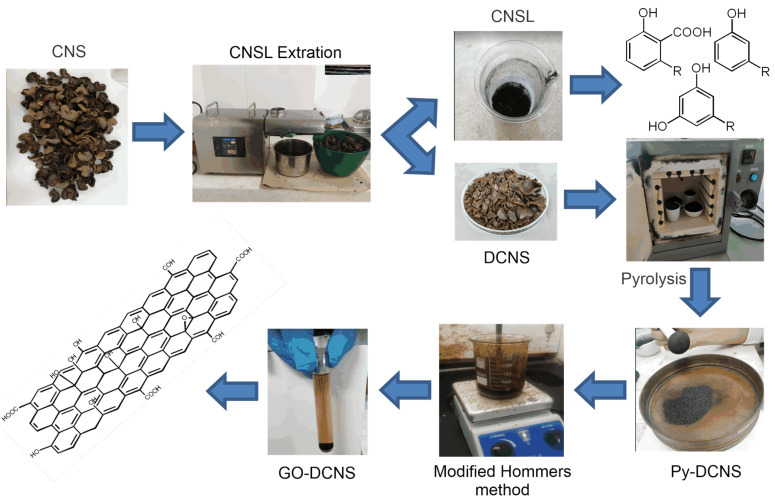
Process for obtaining graphene oxide from cashew nut shell waste.

For the synthesis of graphene oxide, 5 g of DCNS were taken, and the modified Hummers method was used, which consisted of adding 50 mL of sulfuric acid and 1 g of sodium nitrate and kept under constant stirring for 30 min in an ice bath. 3 g of potassium permanganate were added and stirring was continued for 3 h. The ice bath was then removed and stirring was continued for 1 h at 35 °C. Deionized water (50 mL) was then added and stirred for 1 h, maintaining the temperature at 100 °C, then stopping the heating and adding 100 mL of deionized water. The mixture was allowed to rest for 15 min and 10 mL of 30% hydrogen peroxide were added. The mixture was allowed to rest for centrifugation to obtain the graphene oxide precipitate. The mixture was then washed with ultrapure water and dried in an oven at 100 °C for 24 h. Figure 8 shows a diagram of the cashew nut shell transformation process from the moment it enters the laboratory. The diagram shows the different steps of mechanical and chemical transformation of the cashew nut shells until they are converted into graphene oxide.

### 3.4. Characterization of the Obtained Materials

FTIR spectroscopy analysis was performed on samples at room temperature after recording the background of the equipment. A scanning range of 4000 to 400 cm^−1^ was used, with a resolution of 4 cm^−1^ and a mirror speed of 0.4 ${cms}^{-1}$. 100 spectra were taken to average the record. Raman spectroscopy was performed at room temperature with a He-Ne laser excitation light (633 nm) with 0.5 mW power and a spectral resolution of 1 ${cm}^{-1}$. Thermogravimetric analysis was performed after calibration of the equipment with an indium standard. Thermal decomposition curves were recorded with samples of approximately 20 mg collected in ceramic capsules, a heating rate of 10 °C ${min}^{-1}$, a flow of 20 mL min of oxygen and a temperature range of 35 to 980 °C. Morphological characterization was performed via scanning electron microscopy with an energy dispersive detector; the samples were pretreated with a 10 nm gold–palladium coating.

## 4. Conclusions

This study demonstrates the potential of utilizing cashew nut shells (CNS) as a sustainable source for producing graphene oxide (GO). Graphene oxide can be synthesized from biomass from deoiled cashew nut shells using the modified Hummers method. The optimization of the pyrolysis process allowed the establishment of optimal conditions, a temperature of 950 °C and a time of 1.8 h to obtain yields of 29.09% and purity of 82.55%. In addition, the structural analyses performed by FTIR spectroscopy and Raman spectroscopy confirmed the procedures’ effectiveness. The FTIR characterization of the synthesized graphene oxide allowed the establishment of the presence of oxygenated functional groups (CO, COH, COOH, among others) in the carbonaceous structure generated in the chemical transformation, while the Raman spectroscopy showed a characteristic spectrum of this graphene material with the D and G bands and the ratio of their typical intensities. In addition, the spectrum showed a weak 2D band typical of structures with few layers. 

Additionally, GO-DCNS showed the thermal decomposition zones corresponding to this compound with a degradation zone for the oxygenated functional groups and a wide degradation zone for the carbon skeleton. The characterization carried out by SEM-EDX established a laminar and corrugated morphology with a chemical composition made up mainly of carbon and oxygen atoms, most of which are constituents of good-quality graphene oxide. This work’s finding paves the way for further exploration of bio-waste as a source of advanced materials, enhancing graphene oxide synthesis’s sustainability and economic feasibility. This approach addresses waste management issues and contributes to the development of sustainable methods for producing graphene oxide, with broad applications in electronics, energy storage, and biomedicine. The findings support the viability of cashew nut shells as an eco-friendly and cost-effective precursor for graphene oxide production, presenting a promising alternative to conventional raw materials.

## Figures and Tables

**Table 1:** Vibration bands of the FTIR spectra of DCNS, Py-DCNS and GO-DCNS.

Assignments	$\mathrm{Wavenumbers}\;({cm}^{-1}$)		
	DCNS	Py-DCNS	GO-DCNS
OH	3334	−	3356
${sp}^{2}$ CH	3009	−	2951
${sp}^{3}$ CH	2924–2854	−	2906–2848
C=O	1646	−	1719
C=C	−	−	1632
O-H	1604		−
C-H	1450	−	−
C-OH	1153	−	1149
OH	1244	−	−
C-O	1027	−	1030
Ar-C-H	844	−	867

**Table 2:** EDX Elemental Analysis of Py-DCNS and GO-DCNS.

Element	Py-DCNS		GO-DCNS	
	Weight %	Atomic %	Weight %	Atomic %
C	85.5	91.1	53.7	62.4
O	8.3	6.6	40.8	35.5
Co	1.4	0.3	−	−
Na	1.1	0.6	−	−
Mn	−	−	1.4	0.4
S	2.1	0.8	3.2	1.4
K	1.6	0.5	0.9	0.3

## Data Availability

The data that support this work and its results are not available to be shared because they are under confidentiality agreements. Access to the data can be requested through an official document.
